# Surface Bubbles Emergence as an Indicator for Optimal Concrete Compaction

**DOI:** 10.3390/ma17102306

**Published:** 2024-05-13

**Authors:** Hassan Ahmed, Jouni Punkki

**Affiliations:** Department of Civil Engineering, Aalto University, 02150 Espoo, Finland

**Keywords:** concrete, compaction quality, surface bubbles, segregation

## Abstract

Compaction quality significantly influences the strength and durability of concrete in structures. Under-compacting can retain entrapped air, reducing strength, while over-compacting can lead to segregation, creating local variances in strength distribution and modulus of elasticity in the concrete structure. This study examines the widely adopted concept that compaction is optimal when bubbles cease to emerge on the concrete surface. We recorded the surface activity of six comparable concrete specimens during the compaction process using a 4K video camera. Four specimens were compacted using a table vibrator and two with a poker vibrator. From the video frames, we isolated the bubbles for analysis, employing digital image processing techniques to distinguish newly risen bubbles per frame. It was found that the bubbles continuously rose to the surface in all specimens throughout the compaction process, suggesting a need for extended compaction, with some specimens showing a slow in the rate of the bubbles’ emergence. However, upon examining the segregation levels, it was discovered that all the specimens were segregated, some severely, despite the continued bubble emergence. These findings undermine the reliability of using bubble emergence as a principle to stop compaction and support the need for developing online measurement tools for evaluating compaction quality.

## 1. Introduction

The compaction process is essential for ensuring the strength and prolonged durability of concrete [1,2,3,4,5], a building material widely used for its robust properties. Compaction liquifies fresh concrete to fill the molds and removes unwanted entrapped air from the concrete [6,7]. Fresh concrete can contain from 5–20% unwanted entrapped air [7] which reduces the strength of hardened concrete by 5%-point for every 1%-point increase in entrapped air [8]. Moreover, the remaining entrapped air negatively impacts the durability of the concrete [6,7], leading to problems such as honeycombing and exposed reinforcement steel [2].

Entrapped pores can have overlapping characteristics with entrained pores, as mentioned in ASTM C457 [9], which prevents the standard from setting a specific size for entrapped pores. On the other hand, Mehta & Monteiro [10] recognize pores larger than 1 mm as entrapped, whereas the Finnish standard for concrete quality control [11] sets a threshold at 0.8 mm for entrapped pores.

To expel these entrapped bubbles, the concrete is compacted via vibration, in which the bubbles escape the fresh concrete by rising to the surface [6,7,12]. However, longer periods of vibration might cause the concrete to segregate, which results in uneven density distribution and further durability issues [1,4,6,7,13]. Gao et al. [13] found a linear relationship between over-vibration-induced segregation and both compressive strength and chloride ion penetration in concrete. Their findings showed that differences in compressive strengths between the top and bottom parts of the segregated concretes can reach up to 12%. More critically, chloride ion penetration exhibited a substantial discrepancy, with a difference of 72% between the top and bottom, highlighting the significant impact of over-vibration on concrete quality. Hence, compaction by vibration should be controlled to minimize segregation and maximize the expulsion of entrapped air from the concrete.

The effect of vibration time and workability on the amount and distribution of entrapped air in concrete was extensively studied by Ahmed et al. [14]. The authors [14] investigated the pore size distribution, porosity profiles, and sphericity of entrapped pores in three different concrete mixes. They [14] concluded that for less workable concretes, there exist larger concentrations of entrapped pores at certain parts of the concrete, especially for lower vibration times. As for more workable concretes, it was found that although the expulsion of entrapped air was more efficient, those concretes had a higher risk of segregation. These findings reinforce the need for a method to determine the point of optimal compaction during the vibration process.

As per the guidelines in the European standard EN 12390-2, concrete must be vibrated for minimal durations until full compaction has been achieved. However, the standard does not specify how to determine when the point of full compaction is reached [15]. According to the American Concrete Institute’s Guide of Consolidation of Concrete 309R-05, compaction should be stopped when the bubbles stop surfacing [7]. Similarly, both the Australian guide for compaction [6] and the supplementary guide to the British Standard EN-13670-1:2000 [16] convey the same condition regarding the cessation of rising bubbles. Such guidelines lead to the common onsite practice where the decision to stop vibration is based on the operator’s observation of bubbles no longer rising to the surface. [6,7,16]. This introduces a great deal of subjectivity to an important process such as compaction that is determinant to the quality of hardened concrete [3,17]. The subjectivity involved in onsite decisions about when to stop compaction underlines a crucial challenge in concrete quality control. In response, many researchers attempted to develop new methods to objectively track or evaluate the compaction of fresh concrete [1,3,4,17,18,19,20,21].

Tian and Bian [20] devised a method for tracking vibration location and duration, which relied on a global positioning system paired with a real-time kinematics measuring mode. Similarly, Gong et al. [4] tracked the motion of vibrators using ultrawide-band tags and recorded the vibration duration from real-time logging data. Furthermore, Tian et al. [3] developed a system that tracks the location and duration of the vibration, where the first is recorded via a global navigation satellite system, and the latter by calculating the difference in motor voltage. Rather than adhering to the practice of monitoring surface bubble activity, these methods rely on measuring the vibration duration and comparing it with a predetermined optimum value. However, such methods may not accommodate the variable nature of concrete during compaction. Ojala et al. [1] demonstrated this inconsistency, showing that identical vibration times applied to two comparable concrete mixes resulted in segregation in one, but not in the other, highlighting a shortfall of time-based compaction monitoring.

In addition to measuring the vibration duration, Wang et al. [17] implemented an IoT-based solution with the capability to capture and analyze concrete surface images through a fine-tuned ResNet-50 model. Furthermore, Ren et al. [21] improved the surface image analysis process by using semi-supervised learning with data augmentation. In these two methods, compacted concretes were classified as ‘unqualified’, ‘middle’, or ‘qualified’ grades by evaluating the surface appearance. Notably, the initial image labeling, a critical step in model training, was conducted by the researchers, introducing potential bias. Moreover, the classification rationale largely depended on visible surface aggregates, which may not reliably reflect the compaction state.

Ojala et al. [1] investigated the use of AC impedance spectroscopy as a means of monitoring compaction. The bulk resistivity was calculated from the impedance values measured during the process of compaction. In cases of over-compaction, they found a high correlation between the difference in bulk resistivity and the densities of the same concrete layers. While this approach objectively detects segregation caused by over-compaction, it does not provide any limits for when to stop the compaction process.

Li et al. [18] evaluated the quality of vibration based on the distribution of vibration energy in fresh concrete. Furthermore, Li et al. [19] developed the method to incorporate the influence of variables like aggregate proportion and rebar spacing on energy distribution and absorption, enhancing the precision of the vibration quality assessment in reinforced concrete. However, the two methods rely on energy thresholds determined by mathematical calculations and previous experiments. This approach does not consider the heterogeneity of concrete, even if cast from the same batch, which suffers from the same problem as that of time-based compaction monitoring [1].

Kisaku et al. [12] established a method to assess concrete compaction quality by quantifying surface bubbles on hardened concrete, discovering a significant correlation between vibrator acceleration and the surface bubble ratio. However, since this method can only be applied to hardened concrete, it is limited to post-cast analysis and cannot monitor the compaction process in real-time. They also proposed a formula linking acceleration to the surface bubble ratio across various concrete mixes, using rheological constants derived from slump values. This approach can oversimplify the complex behavior of concrete by relying on generalized estimates rather than real-time measurements.

Given the challenges and limitations associated with these alternative methods, the traditional practice of observing bubble movement during compaction retains its relevance in the field. However, the assumption that optimum compaction is achieved once the bubbles cease to rise to the surface remains largely unverified and may hold implications for the overall quality and durability of the hardened concrete. Hence, this article focuses on evaluating the reliability of using bubble cessation as an indicator to stop concrete compaction. To this end, we recorded the concrete surface during compaction using a 4K video camera. By analyzing frames with digital image processing (DIP), we examined the properties and behavior of the rising bubbles. Furthermore, we investigated the level of segregation [14,22] of each of the concrete specimens to test the impact of relying on bubble cessation on the quality of compacted concrete.

## 2. Materials and Methods

### 2.1. Materials and Mix Design

In this research, all concrete specimens were cast using a mix that incorporated CEM II/B-M (S-LL) 42.5 N, produced by Finnsementti in Parainen, Finland. The chemical composition of the clinker is detailed in Table 1. The cement composition includes a mixture of 11% limestone and 17% ground granulated blast-furnace slag.

The concrete mixes contained a blend of granitic gravel and white limestone aggregates each exhibiting a water absorption rate of 0.8%. The granitic gravel was divided into six distinct fraction sizes, while the white limestone was used in a single fraction size, notably the largest at 8–16 mm. The distribution and sizes of these aggregates are detailed in Table 2. Regarding specific gravities, the values were 3.15 for cement, 2.68 for granitic gravel, and 2.72 for white limestone.

The composition of the mixtures was measured as 430 kg/m^3^ of cement, 170 kg/m^3^ of water–accounting for absorption by the aggregates, and 1703 kg/m^3^ of aggregates, resulting in an effective water-to-cement ratio of 0.395. Air entrainment was achieved with the addition of BASF MasterAir 100, sourced from Finland, to reach an average air content of 6.0%, and workability was enhanced through the incorporation of the superplasticizer BASF MasterGlenium SKY 600, also from Finland. The doses for the air-entraining agent and superplasticizer were set at 0.025% and 0.77% of the cement weight, respectively, aiming for an S4 slump class in the concrete.

### 2.2. Methods

#### 2.2.1. Concrete Mixing, Standard Testing, and Casting

Two concrete batches were prepared in a pan-type mixer and each mixed for three minutes and 30 s at a controlled ambient temperature of 20 ± 2 °C. After mixing, the slump of each batch was assessed in alignment with SFS-EN 12350-2 standards [23], followed by the measurement of air content using the pressure method, as mentioned in SFS-EN 12350-7 [24]. If the slump measurement fell outside the S4 range of 160–210 mm or if the air content deviated from the 6.0 ± 1.0% range, the batch was considered unacceptable and subsequently discarded. The process was repeated, mixing new batches until both slump and air content specifications were satisfactorily met. The first accepted batch was cast into four cylindrical metallic molds, whose height is 300 mm, and their inner diameter is 150 mm. On the other hand, the second batch was cast into two box-type wooden molds measuring 200 mm in length, 200 mm in width, and 300 mm in height. Casting in both mold types was done in one layer and kept as consistent as possible.

#### 2.2.2. Compaction and Surface Video Recording

The compaction of the four concrete samples cast into the cylindrical molds was achieved using the high-frequency vibrating table 2.0271SU at a frequency of 150 Hz and an amplitude of 0.2 mm. On the other hand, the remaining two concrete samples, poured into box-type molds, underwent compaction via the poker vibrator Wacker Neuson IRFU 30/5. This vibrator is characterized by a 350 mm long head and a 30 mm diameter, operating at a vibration frequency of 200 Hz and an amplitude of 1 mm, which generates an effective vibration diameter of 400 mm.

Table 3 outlines the dimensions of the molds, vibration methods, and corresponding specimen names for each mold type. Specimens from the cylindrical molds, compacted by the table vibrator, are denoted by a ‘T’ prefix, signifying ‘Table vibrator’, and are followed by their specific vibration durations in seconds. This group will be referred to as the ‘T-group’. Similarly, specimens from the box-type molds, compacted with the poker vibrator, carry a ‘P’ prefix, indicative of ‘Poker vibrator’, followed by their vibration durations in seconds, and will be known as the ‘P-group’. For example, the specimen named ‘T140’ represents a table-vibrated specimen compacted for 140 s, while ‘P35’ corresponds to a poker-vibrated specimen compacted for 35 s.

**Figure 1:**
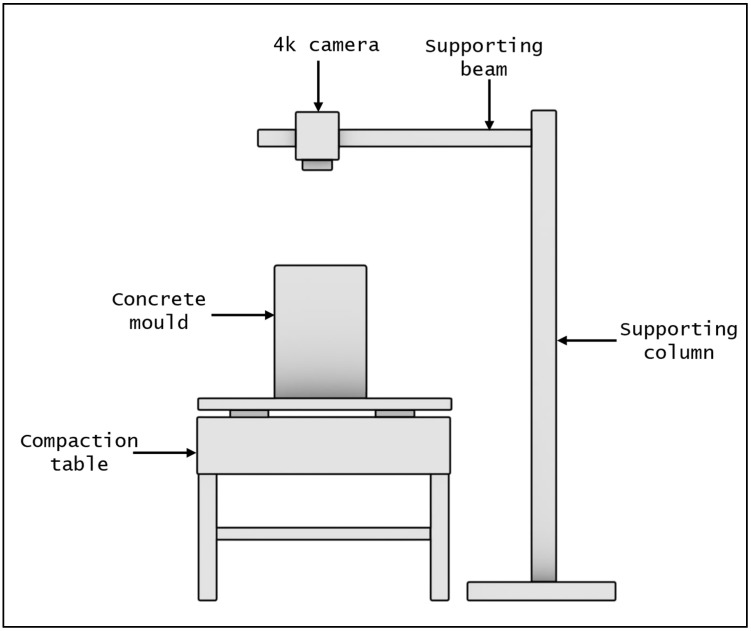
Schematic representation of the test setup for the T-group. For the P-group, the vibrating table was turned off and the poker table was inserted into the mold.

During compaction, the surface of the concrete was video recorded using a Sony FDR-AX100E 4k Camcorder purchased in Helsinki, Finland. The video camera was securely mounted onto a horizontal steel beam supported by a steel column, as illustrated in Figure 1. The camera was positioned at a fixed distance of 50 cm from the concrete surface, ensuring that the entire surface of each mold fits within the frame and maintaining consistency in the recording across all videos. Furthermore, uniform lighting conditions were maintained throughout the recording process to ensure consistency in the videos. All videos were captured in 4k quality, chosen for their high resolution to capture the activity of surfacing bubbles. A frame rate of 25 fps was employed, and the footage was saved in .MP4 format for subsequent processing.

#### 2.2.3. Frames Extraction and Bubbles Selection

A Python script was employed to extract frames from the recorded videos. Frames for the T-group were extracted at five-second intervals, while for the P-group, frames were extracted every one second. The decision to use longer intervals for the T-group was based on their extended compaction times and the infrequent changes observed on the surface bubbles during compaction. In contrast, the shorter compaction times of the P-group necessitated more frequent frame extractions. All extracted frames were saved as .PNG files for the process bubbles selection.

**Figure 2:**
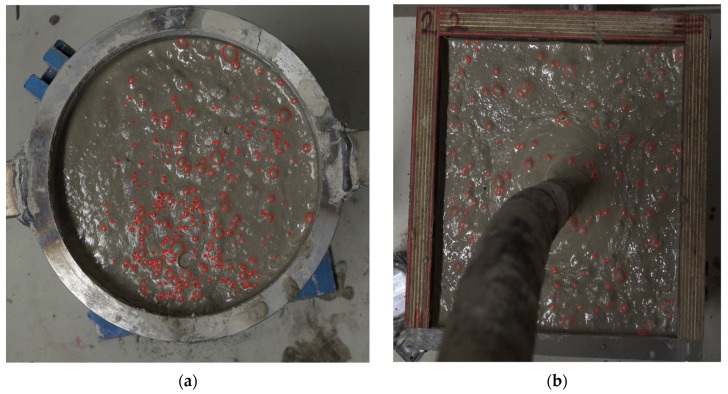
Example frames with selected bubbles in red using the MATLAB application developed by Haas et al. [25]: (**a**) frame at 75 s from video T80; and (**b**) frame at 29 s from video P35.

Bubbles in the frames were selected using a MATLAB R2021a application designed by Haas et al. [25] as shown in Figure 2. This application was developed for selecting bubbles in gas-liquid multiphase flows. To select the bubbles, an image or set of images is loaded into the application and the bubbles are marked as ellipses onto each image by dragging and dropping ellipses. Once the selection is complete, the data is saved in a .mat file. In this research, the frames from each video were loaded at once, and as the selection was completed for all the frames, they were saved into one .mat file. Those files contain the data of each image and its overlayed ellipses per each video. For the images, the resolution and pixel values are stored, whereas for the ellipses, it saves their center coordinates, semi-axes, rotation angles, and vertices.

#### 2.2.4. Sawing and Density Measurements

All the concrete specimens were left to harden in a controlled temperature of 20 ± 2 °C for 24 h, after which they were demolded. In line with the density-based approach for evaluating segregation in concrete as detailed by Ahmed and Punkki [22], the specimens were then sawn into 10 horizontal slices, each about 25 mm thick, using a diamond-blade saw. The density of each slice was measured in accordance with the standard EN 12390-7 [26], involving weighing the slices both in air and underwater and calculating the density as the weight in air divided by the difference between the air and underwater weights. The density-based segregation index [22] was determined based on the standard deviation of these density values across the 10 slices.

## 3. Results and Analysis

### 3.1. Fresh Concrete Properties

The workability and air content results for both groups are shown in Table 4. Both groups had similar slump values, where the slump of the P-group was only 5 mm higher than that of the T-group, and both are classified as class S4. For the air content, the P-group had a 0.6% increase in air over that of the T-group, but both fall within the previously specified range of 6.0 ± 1.0%, which represents a commonly used value for air entrainment in the concrete industry.

### 3.2. Surface Bubbles Analysis

#### 3.2.1. Initial Processing of Bubbles Data

To analyse the selected bubbles in the .mat files, we loaded the data into Python using the ‘scipy.io.loadmat’ function. This step imported the data as a ‘void’ array, a specialized structure in Python designed to handle the complex, heterogeneous data obtained from the MATLAB processing. Unlike conventional arrays, this ‘void’ array format encapsulates a rich dataset for each video frame, organizing it into a structured form, which preserves the attributes of the frames and the selected bubbles on each frame. This includes, but is not limited to, arrays of bubble centre coordinates and their semiaxes lengths.

In this study, we follow the limits set by the Finnish concrete industry, which establishes a minimum diameter of 0.8 mm for entrapped air pores, commonly used in pores analysis. Accordingly, our dataset excludes bubbles with an equivalent diameter smaller than 0.8 mm. This exclusion is based on calculating the equivalent diameter for each bubble, represented as an ellipse, and removing those not meeting the size criterion from the dataset arrays.

**Figure 3:**
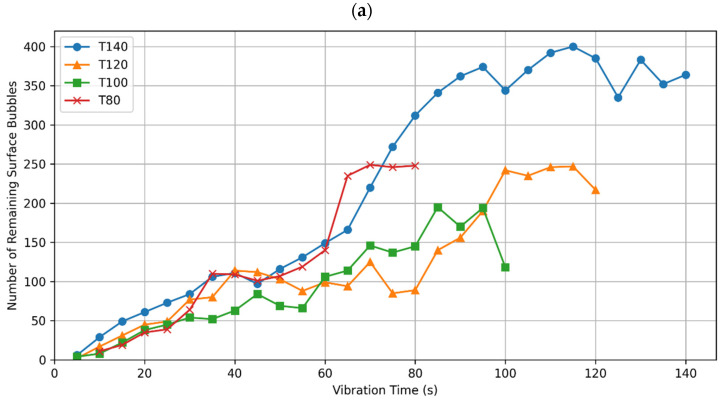

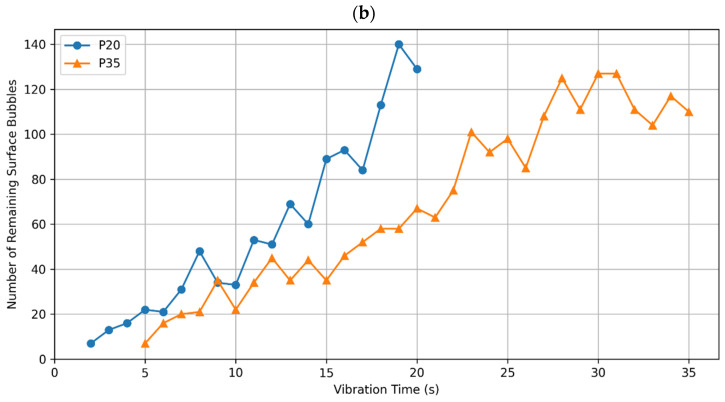
Number of selected bubbles that remain on the surface in each frame, plotted against the vibration time for: (**a**) T-group; and (**b**) P-group specimens.

It is worth noting that the selected bubbles represent those that remained on the surface during the process of compaction, and are not solely the newly risen bubbles, which will be analysed in the following subsection. Bearing that in mind, Figure 3a,b show the change in the number of surface bubbles across the vibration time for the T-group, and P-group, respectively.

During the compaction process, it was a common occurrence for many bubbles to rise to the surface and remain there without bursting. This phenomenon was notably more pronounced in the T-group than in the P-group, as evidenced in the example frames shown in Figure 2. Specifically, the surface of the T80 specimen appears significantly more populated with bubbles compared to that of the P35 specimen. This observation is quantitatively supported by the data presented in Figure 3, where, for example, the T80 surface hosts approximately 250 bubbles, more than double the count observed on the surface of P35.

Similarly, when comparing other specimens from the T-group with those from the P-group, a consistently higher number of bubbles remaining on the surface is observed in the T-group specimens. This discrepancy can largely be attributed to their extended compaction times in comparison to those of the P-group. Moreover, the variance in compaction methods plays a critical role; the T-group specimens are compacted using a vibration source positioned at the bottom, leading to a relatively undisturbed surface. In contrast, the P-group specimens are compacted with a poker vibrator, resulting in more vigorous surface agitation. This difference in compaction dynamics is apparent in the video recordings of the process.

#### 3.2.2. Newly Risen Bubbles per Frame

Since the focus of this article is testing the validity of bubble cessation as a criterion for concluding the concrete compaction process, it is essential to differentiate newly risen bubbles from those persisting across consecutive frames. Given the manual bubbles selection process frame by frame, there is a possibility of reselecting the same bubble across a pair of frames, potentially representing it twice in the dataset with slightly varied geometric values. To mitigate this, we devised a strategy to discern ‘old’ from ‘new’ bubbles in successive frames. Bubbles persisting from frame ‘i’ to ‘i + 1’ exhibit minimal displacement and maintain similar dimensions, evidenced by their center coordinates and semiaxes lengths. Thus, by identifying bubbles with minimal changes as ‘old’, we can focus our analysis on the ‘newly’ risen bubbles.

Between frames ‘i’ and ‘i + 1’, those old bubbles would have very similar center coordinates since they would have moved only slightly in either x or y directions or both, while at the same time having almost the same size, which can be translated as similar values for their semiaxes. Consequently, other bubbles are considered newly risen, and we can exclude all old bubbles from our analysis. Hence, the next step is to identify tolerances for displacements in the x and y directions and changes in size for both semiaxes.

To quantify the tolerances for identifying ‘old’ bubbles, we manually tracked the changes in the properties of six bubbles across three pairs of consecutive frames for each video. This process involved manually tracking 24 bubbles for the T-group and 12 for the P-group. The tolerances for each group are determined as the average value of changes in each selected property.

For the T-group, we calculated movement tolerances at Δx = Δy = 6 mm, to account for the maximum allowable horizontal and vertical shifts between frames. In contrast, for the P-group, a less stringent tolerance of Δx = Δy = 9 mm was found, due to the slightly more aggressive movement of the surface while compacting with a poker vibrator. Regarding the tolerance for changes in the semiaxes lengths, the average value was determined to be 1 mm for the T-group and 2 mm for the P-group.

**Figure 4:**
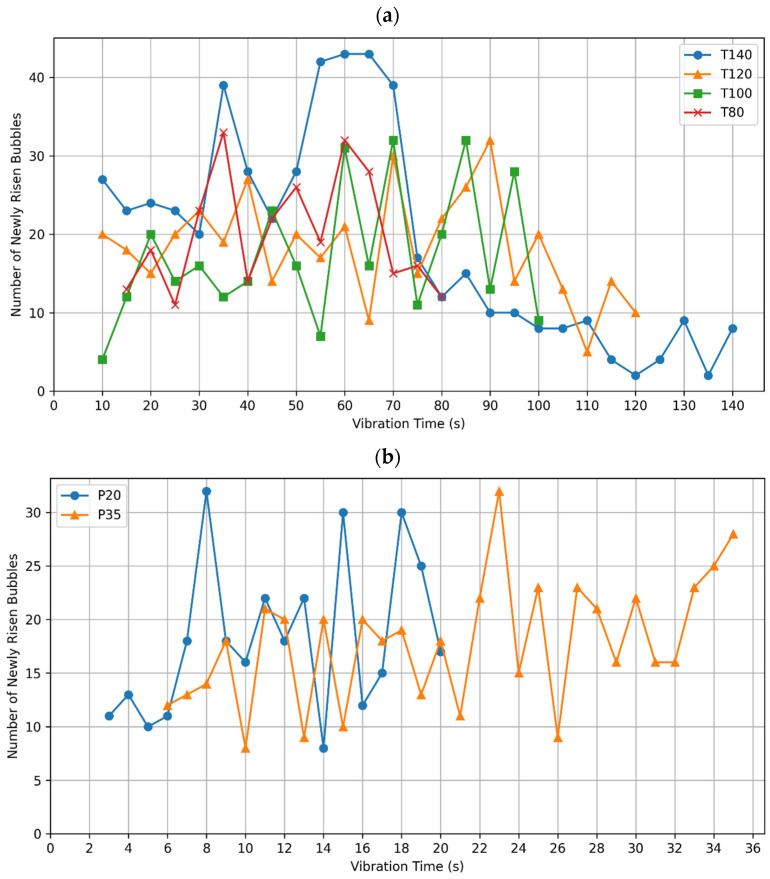
Number of newly risen bubbles in each frame, plotted against the vibration time for (**a**) T-group; and (**b**) P-group specimens.

Figure 4a,b represent the number of newly risen bubbles plotted against the vibration time for the T-group and P-group, respectively. A significant plummet in the number of bubbles is found for all specimens when compared to their counterparts with all remaining bubbles on the surface shown in Figure 3. For the T-group, an average of 14% of surface bubbles were classified as new, while the rest were removed from the analysis, with T140 showing the highest concentration of old bubbles of 92%. On the other hand, P20 and P35 had 30% and 35% of the selected bubbles classified as new.

As shown in Figure 4a, bubbles continue to emerge in specimens T80, T100, and T120 throughout the compaction process, with the rate of emergence exhibiting general fluctuations. T140, however, displays a distinctly different pattern, with a marked transition in the rate of bubble emergence at the 70-s mark. Initially, T140 shows a higher frequency of bubbles, which significantly decreases after 70 s, suggesting that a large part of compaction air was expelled during the first 70 s. As for the bubble activity in P20 and P35, shown in Figure 4b, there are consistent fluctuations in bubble emergence throughout the compaction process.

#### 3.2.3. Patterns of Bubbles Rising Behavior

**Figure 5:**
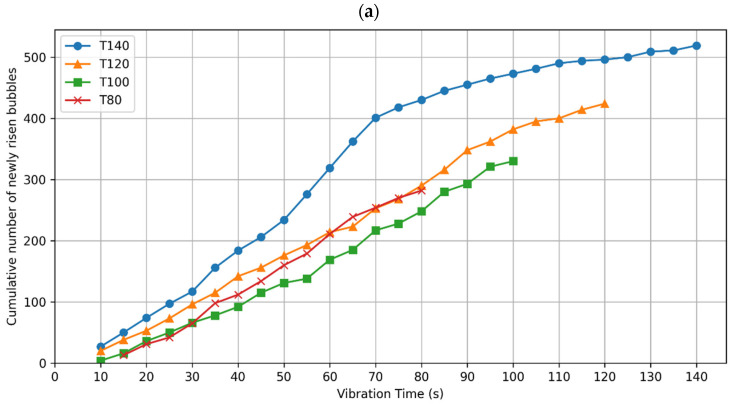

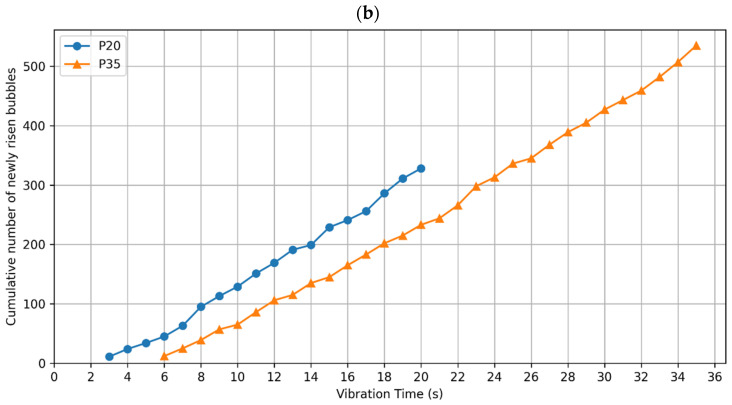
Cumulative number of newly risen bubbles in each frame, plotted against the vibration time for (**a**) T-group; and (**b**) P-group specimens.

To test the hypothesis that optimal compaction correlates with a cessation of new bubbles surfacing, we conducted a cumulative bubble analysis over the compaction frames. If the bubbles cease rising, the cumulative bubble count line should be horizontal, denoting a slope of zero. We plotted the cumulative number of newly risen bubbles against compaction time for both the T-group and P-group specimens, as illustrated in Figure 5a,b, respectively. It is shown in Figure 5 that the slope of the lines is never zero, which denotes that the bubbles continuously rose to the surface in all specimens. According to the bubble cessation rule, this would mean that all these concretes should have been compacted for a longer time.

Upon closer inspection, Figure 5a reveals some slope reductions for T140, T120, and T80 at 70 s, 85 s, and 65 s, denoted as deceleration points in Table 5. On the contrary, the slopes hardly change for T100, P20, and P35, highlighting a continuous rise of bubbles to the surface without slowing. The decrease in the slopes suggests a slowdown in the rate of bubbles rising to the surface.

To quantify these changes numerically, we divided the cumulative plots for T140, T120, and T80 into two segments: one prior to the deceleration point and one following it. Linear regression was applied to each segment to calculate the slopes, representing the bubble emergence rates at each stage. The initial segments showed very strong fits with R^2^ values of 0.99 for all three specimens, while the post-deceleration segments also demonstrated high fits with R^2^ values of 0.95 for T140, 0.98 for T120, and 0.96 for T80. These calculated slopes are presented in Table 5 as the ‘initial’ and ‘decreased’ rates in bubbles per second.

Investigating the initial rates shown in Table 5. T140 had the highest initial rise rate in bubbles of 6.2 bubble/s, almost 55% and 38% quicker than those of T120 and T80, respectively. This very high initial rate of T140 is followed by the lowest decreased rate of 1.6 bubbles/s, which is about 65% of that of T120, and about 55% of that of T80. This shows that even though there is a significant decrease in the rate of bubbles rising for T140, it was preceded by a much higher rate of bubbles rising compared to all other specimens.

As for the difference in the bubbles’ rising rates for each specimen before and after the deceleration point, T140 exhibits the most significant change with a decrease of 4.6 bubbles/s, while T120 and T80 show more modest reductions of 1.5 and 1.6 bubbles/s, respectively. Such subtle changes would be challenging to detect with the naked eye on-site. Furthermore, since none of the slope reductions approach zero, this indicates that the bubbles continued to rise, suggesting that compaction should have been extended if relying solely on the cessation of bubbles as an indicator for optimal compaction.

### 3.3. Segregation Analysis

**Figure 6:**
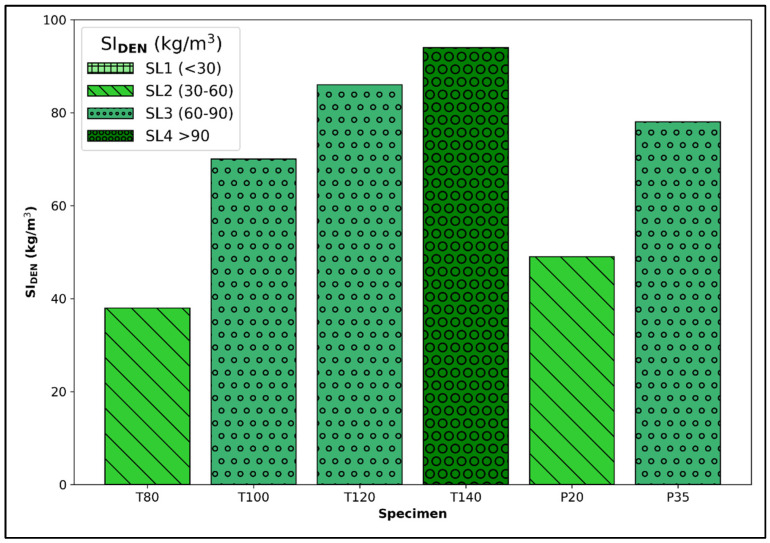
Standard deviation in densities across the ten slices, denoted by SIDEN, alongside the related levels of segregation (SL1, SL2, SL3, and SL4) and their boundaries, for T- and P-group specimens.

The density-based segregation index, SIDEN, was calculated for each specimen to evaluate its level of segregation in accordance with the methodology outlined by Ahmed and Punkki [20]. SIDEN is defined as the standard deviation of the densities of 10 sawn discs from each specimen. The SIDEN values for both the T- and P-groups are illustrated in Figure 6. Furthermore, Ahmed and Punkki [20] identified four segregation levels (SL): SL1, SL2, SL3, and SL4, based on specific ranges detailed in the legend of Figure 6. As depicted, none of the specimens from either the T- or P-group fell into the lowest segregation category, SL1, indicating that all specimens exhibited some degree of segregation.

For the T-group, T80 exhibited the lowest level of segregation (SL2) with a SIDEN value of 38 kg/m^3^. Both T100 and T120 were categorized within the SL3 range, with SIDEN values of 70 and 86 kg/m^3^, respectively. The highest segregation index, 94 kg/m^3^, was observed in T140, placing it in the highest segregation category, SL4. In contrast, within the P-group, P20 was classified under SL2 with a SIDEN of 49 kg/m^3^, while P35, with a SIDEN of 78 kg/m^3^, was categorized within SL3.

As explained by the authors [20], those assigned segregation levels are suggestions and the criteria for accepting or rejecting a concrete due to its high segregation level will largely depend on the application of the structural element made of that segregated concrete. Despite these variances in application, it is evident that all specimens in our study exhibited significant variations in density within each specimen, categorizing them as segregated. This indicates a shortfall in compaction quality; optimal compaction aims to minimize segregation, a goal not achieved in the specimens studied, especially evident for T100, T120, T140, and P35.

## 4. Discussion

Our analysis of the surface bubbles across the six tested concretes reveals continuous bubble emergence during compaction for all the specimens. This finding challenges the effectiveness of using bubble cessation as a marker for optimal compaction time. Despite ongoing bubble activity, all specimens exhibited some degree of segregation; notably, T140 showed the most severe segregation, followed by T120, P35, and T100, while P20 and T80 showed the least. These results underscore that relying solely on bubble emergence as a cue to halt compaction could lead to significant segregation.

Furthermore, there are variations in the profiles of emerging bubbles as presented in Figure 5a. Even though the T-group specimens were cast from the same concrete batch, and compacted by the same table vibrator, they displayed three different behaviors of bubbles emergence. Firstly, T100 showed a continuous rise in bubbles at a similar rate throughout the whole compaction time. Secondly, T140 showed the highest initial rate of bubbles rising until 70 s of compaction, compared to its remaining three counterparts, which was followed by the largest plummet as well. Lastly, T120 and T80 showed similar initial rates to each other and similar minor drops in these rates. Such variance among very similar specimens supports the notion that observing emerging surface bubbles can be a non-reliable method for achieving optimal compaction.

It is worth noting that our study has some limitations, such as studying one air-entrained mix with a high workability, which is more prone to segregation than stiffer mixes or those without air entrainment. Future research could broaden this scope by examining other concrete mixes, such as non-air entrained concretes, less workable concretes, or semi-self-compacting concretes that require minimal vibration efforts. Such investigations could offer a more comprehensive understanding of bubble dynamics in different concretes. Additionally, the manual identification of bubbles in each frame, while thorough, is time intensive. Future studies might leverage machine learning models to automate bubble detection, significantly enhancing efficiency. Furthermore, future research should focus on real-time measurements to evaluate the quality of compaction rather than solely relying on observing surface bubbles.

## 5. Conclusions

Compaction is a critical process that affects the strength and durability of concrete, traditionally guided by the principle of stopping the process once bubbles cease to surface, indicating supposed optimal compaction. This research investigated that principle by capturing videos of concrete specimens during the compaction process and analyzing the emergence of new bubbles to the surface throughout the compaction time. Afterwards, the segregation levels of those specimens were assessed through density measurements to evaluate their compaction quality.

The investigation revealed the following.

1. None of the six tested specimens exhibited a total cessation of bubble emergence, indicating the need for prolonged compaction. However, all the specimens exhibited various levels of segregation, with some more pronounced than others.
2. Half of the specimens demonstrated continuous bubble emergence without any signs of slowing.
3. The remaining three specimens showed a decrease in bubble emergence rates, yet none approached a cessation, with the lowest rates recorded at 1.6 and 2.5 bubbles/s.
4. Despite being cast from the same batch and compacted by the same compaction table, the specimens displayed differing bubble emergence profiles.

These findings suggest that relying on bubbles no longer rising to the surface does not work as a suitable method for optimizing compaction quality, as it could lead to heavily segregated concretes, which compromises the quality of the concrete. Hence, our findings point towards the need for developing more online focused measurements during the compaction process rather than solely relying on the emergence of surface bubbles.

## Figures and Tables

**Table 1:** Chemical composition of the clinker of CEM II/B-M (S-LL) 42.5 N.

Chemical Composition	Mass Percentage Volume (%)
CaO	64–66
SiO_2_	20–22
Al_2_O_3_	4.0–5.4
Fe_2_O_3_	2.8–3.2
MgO	2.5–3.2
SO_3_	3.0–3.3

**Table 2:** Combined aggregates sizes and their weight proportions of the total aggregates in the concrete mixture.

Aggregate Type	Fraction (Diameter in mm)	Weight Proportion (%)	Sieve Size (mm)									
			0.125	0.25	0.5	1.0	2.0	4.0	8.0	16.0	32.0	64.0
Filler	<1	8	42	81	93	97	98	100	100	100	100	100
Fine Aggregates (FA)	0.1–0.6	12	3	21	76	100	100	100	100	100	100	100
	0.5–1.2	12	0	2	6	70	100	100	100	100	100	100
	1.0–2.0	15	0	1	2	7	79	100	100	100	100	100
	2.0–5.0	15	0	0	1	1	1	47	100	100	100	100
Coarse Aggregates (CA)	5.0–10.0	18	0	0	0	0	0	3	82	100	100	100
	8.0–16.0	20	0	0	0	0	0	0	5	99	100	100
Combined Aggregates (%)			4	9	18	29	44	55	78	100	100	100

**Table 3:** Specimen overview: Mold types, dimensions, vibration methods, and naming conventions indicating vibration time in seconds.

Group	Mold Type	Mold’s Dimensions	Vibration Method	Specimen
T-group	Cylindrical	Ø150 × 120	Table vibrator	T140, T120, T100 & T80
P-group	Box-type	200 × 200 × 300	Poker vibrator	P20 & P35

**Table 4:** Slump values, slump classes, and air content for the T- and P-groups.

Group	Slump (mm)	Slump Class	Air Content
T-group	190	S4	6.1%
P-group	195	S4	6.7%

**Table 5:** Deceleration points and bubble emergence rates before and after deceleration for T140, T100, and T80 covering an area of 176 cm^2^.

Specimen	Deceleration Point (s)	Initial Rate (bubble/s)	Decreased Rate (bubble/s)
T140	70	6.2	1.6
T120	85	4.0	2.5
T100	65	4.5	2.9

## Data Availability

The raw data supporting the conclusions of this article will be made available by the authors on request.
